# Transcriptional Feedback Loops in the Caprine Circadian Clock System

**DOI:** 10.3389/fvets.2022.814562

**Published:** 2022-04-11

**Authors:** Dengke Gao, Hongcong Zhao, Hao Dong, Yating Li, Jing Zhang, Haisen Zhang, Yu Zhang, Haizhen Jiang, Xiaoyu Wang, Aihua Wang, Yaping Jin, Huatao Chen

**Affiliations:** ^1^Department of Clinical Veterinary Medicine, College of Veterinary Medicine, Northwest A&F University, Xianyang, China; ^2^Key Laboratory of Animal Biotechnology of the Ministry of Agriculture and Rural Affairs, College of Veterinary Medicine, Northwest A&F University, Xianyang, China; ^3^Department of Preventive Veterinary Medicine, College of Veterinary Medicine, Northwest A&F University, Xianyang, China

**Keywords:** circadian clock, ruminants, caprine, goat embryonic fibroblasts, BMAL1, NR1D1

## Abstract

The circadian clock system is based on interlocked positive and negative transcriptional and translational feedback loops of core clock genes and their encoded proteins. The mammalian circadian clock system has been extensively investigated using mouse models, but has been poorly investigated in diurnal ruminants. In this study, goat embryonic fibroblasts (GEFs) were isolated and used as a cell model to elucidate the caprine circadian clock system. Real-time quantitative PCR analysis showed that several clock genes and clock-controlled genes were rhythmically expressed in GEFs over a 24 h period after dexamethasone stimulation. Immunofluorescence revealed that gBMAL1 and gNR1D1 proteins were expressed in GEFs, and western blotting analysis further verified that the proteins were expressed with circadian rhythmic changes. Diurnal changes in clock and clock-controlled gene expression at the mRNA and protein levels were also observed in goat liver and kidney tissues at two representative time points *in vivo*. Amino acid sequences and tertiary structures of goat BMAL1 and CLOCK proteins were found to be highly homologous to those in mice and humans. In addition, a set of goat representative clock gene orthologs and the promoter regions of two clock genes of goats and mice were cloned. Dual-luciferase reporter assays showed that gRORα could activate the promoter activity of the goat *BMAL1*, while gNR1D1 repressed it. The elevated pGL4.10-gNR1D1-Promoter-driven luciferase activity induced by mBMAL1/mCLOCK was much higher than that induced by gBMAL1/gCLOCK, and the addition of gCRY2 or mPER2 repressed it. Real-time bioluminescence assays revealed that the transcriptional activity of *BMAL1* and *NR1D1* in goats and mice exhibited rhythmic changes over a period of approximately 24 h in NIH3T3 cells or GEFs. Notably, the amplitudes of *gBMAL1* and *gNR1D1* promoter-driven luciferase oscillations in NIH3T3 cells were higher than those in GEFs, while *mBMAL1* and *mNR1D1* promoter-driven luciferase oscillations in NIH3T3 cells had the highest amplitude. In sum, transcriptional and translational loops of the mammalian circadian clock system were found to be broadly conserved in goats and not as robust as those found in mice, at least in the current experimental models. Further studies are warranted to elucidate the specific molecular mechanisms involved.

## Introduction

Earth's rotation around its axis causes predictable circadian changes in environmental conditions, such as light and temperature. To anticipate and adapt to the rhythmic changes in the geophysical environment, physiological phenomena in living organisms, such as metabolism, body temperature, and neural activity, exhibit rhythmic changes over an approximately 24-h cycle (1, 2). These rhythmic changes are defined as circadian rhythms, and the underlying molecular mechanism generated by living organisms to anticipate and adapt to circadian changes is known as the circadian clock system. Circadian clock systems are ubiquitous in simple single-cell organisms, higher plants, and animals (3, 4). Briefly, the mammalian circadian clock system can be divided into the central circadian clock in the suprachiasmatic nucleus (SCN) of the hypothalamus and peripheral circadian oscillators in almost all tissues and organs of the body (5). The primary circadian pacemaker in the SCN of the mammalian brain is photoentrained by light signals from the eyes through the retinohypothalamic tract, which synchronizes the body's circadian clock system with changes in light and darkness of the external environment. The SCN then transmits phase information to other areas of the brain, peripheral tissues, and organs through a combination of nerves, body fluids, and other signaling pathways, coherently driving the rhythmic oscillations of the peripheral circadian clocks.

At the molecular level, the mammalian circadian clock system is formed by the interconnection of positive and negative transcriptional and translational feedback loops, composed of a set of core clock genes and their encoded proteins (6). To date, more than 20 clock genes have been discovered, including aryl hydrocarbon receptor nuclear translocator-like protein 1 (*ARNTL*, also known as *BMAL1*), circadian locomotor output cycles kaput (*CLOCK*), periods (*PERs, PER1*/*PER2*/*PER3*), cryptochromes (*CRYs, CRY1*/*CRY2*), nuclear receptor subfamily 1 group D member 1 (*REV-ERB*α, also known as *NR1D1*), nuclear receptor subfamily 1 group D member 2 (*REV-ERB*β, also known as *NR1D2*), and retinoid-related orphan receptor alpha (*ROR*α) (7). The peripheral and central circadian clocks share similar molecular mechanisms. The core molecular gear of the circadian clock system includes two transcriptional activators, CLOCK and BMAL1, which heterodimerize and bind to E-box enhancer elements on the promoters of the *PER* and *CRY* genes to activate their transcription (8–10). A few hours later, PER and CRY proteins accumulate in the cytoplasm and are transferred into the nucleus to directly inhibit the transcriptional activity of BMAL1/CLOCK, thus repressing their expression through a negative feedback loop (11). The nuclear receptors REV-ERBα and RORα are also used in complementary mechanisms for maintaining the 24-h rhythmic oscillations of the circadian clock. BMAL1/CLOCK promotes the transcription of *REV-ERB*α and *ROR*α through the E-box cis elements, whereas REV-ERBα and RORα directly bind to the specific retinoic acid-related orphan receptor response elements (ROREs) on the *BMAL1* promoter, which in turn inhibit and promote *BMAL1* transcription, respectively (12–14). In addition, casein kinase CK1ε/δ modifies the PER protein by phosphorylation, which is also involved in the maintenance of circadian rhythms (15). BMAL1/CLOCK also activates the expression of the D site of albumin promoter binding protein (*DBP*) gene by binding to the E-box cis elements within the promoter region (16). REV-ERBα and RORα compete for RORE and control the expression of the nuclear factor interleukin 3-regulated protein (*NFIL3*, also known as *E4BP4*) gene (17, 18). DBP and NFIL3 also control *ROR*α gene expression by binding to the D-box *cis* elements in the promoter regions (19). Simultaneously, BMAL1/CLOCK also activates the transcription of numerous downstream clock-controlled genes by adhering to the E-box, thus regulating a number of physiological functions, such as energy metabolism (20, 21), reproductive capacity (22–24), cell apoptosis (25), cancer (26), and immunoendocrine signals (27, 28).

Mouse models have been at the forefront of research on the circadian clock system owing to easy access to genetic information and relatively easy transgene, targeted gene deletion, and genome-wide random mutagenesis screening. However, few studies have investigated the caprine circadian clock system. The regulation mechanisms of growth, development, and seasonal reproduction of goats have always received much attention owing to their agricultural importance (29). In addition, most studies on the circadian clock have been performed in nocturnal rodents, such as mice, whereas goats are diurnal animals (30–32). Goats are phylogenetically distant from mice, which makes them an interesting animal model. Therefore, delineating the molecular regulatory mechanism of the goat circadian clock will be the key to further dissect and understand the involvement of the circadian clock system in the regulation of physiological functions in seasonal mammals.

In this study, the mRNA and protein expression levels of core clock genes were analyzed using real-time quantitative PCR (qPCR) and western blotting (WB) in goat embryonic fibroblasts (GEFs) *in vitro* and goat liver and kidney tissues *in vivo*, respectively. Subsequently, a set of representative goat clock gene orthologs (*BMAL1, CLOCK, CRY2, ROR*α, and *NR1D1*) and the promoters of two clock genes (*BMAL1* and *NR1D1*) of goats and mice were cloned. A dual-luciferase reporter assay and real-time monitoring of bioluminescence were performed to investigate the salient transcriptional features of the goat circadian clock.

## Materials and Methods

### Animals

Goat fetuses were obtained from pregnant goats at 30 days of gestation (Experimental Goat Farm at Northwest A&F University, Yangling, Shaanxi, China) by cesarean section under sterile conditions. In addition, six clinically healthy male goats (Sannen goat breed, 18 months old, mean body weight 70.5 ± 3.2 kg) were used in this study. All animals were housed in an indoor pen at the experimental goat farm (longitude: 108°4′15″E, latitude 34°17′11″N) of Northwest A&F University over the study period in February 2022, under the local natural photoperiod (sunrise at 07:15 h and sunset at 18:40 h), environmental temperature (approximately 20–25°C), and relative humidity (approximately 40–60%). As per standard farming practices, hay and water were available *ad libitum*. The goats were euthanized by injecting an intravenous overdose of pentobarbital sodium (90 mg/kg BW) at the indicated times (10:00 and 22:00, *n* = 3/time point). Goat liver and kidney tissues were collected immediately and stored at −80°C for subsequent total RNA and protein extraction. All animal procedures were approved under the control of the Guidelines for Animal Experiments by the Committee for the Ethics on Animal Care and Experiments of Northwest A&F University (Approval No. 2021028) and performed under the control of the “Guidelines on Ethical Treatment of Experimental Animals” (2006) No. 398 set by the Ministry of Science and Technology, China.

### Isolation of GEFs and Cell Cultures

GEFs were isolated from 30-day gestational goat fetuses using the tissue block attachment method. Briefly, the fetus was sterilized with 75% ethanol for 1 min and then rinsed with phosphate-buffered saline (PBS). The limbs, head, and viscera of the fetus were removed, and the remaining tissues were rinsed again with PBS and cut into pieces of one mm^3^. Tissue pieces were evenly distributed in Petri dishes. The petri dish was placed upside down in an incubator with a humidified atmosphere of 95% air and 5% CO_2_ at 37 °C. After culturing for 2 h, Dulbecco's Modified Eagle Medium (DMEM)/F-12 supplemented with 10% fetal bovine serum (FBS, Gibco, Carlsbad, CA, USA) and 1 × antibiotic-antimycotic (AA, containing penicillin, streptomycin, and amphotericin B; Invitrogen, Carlsbad, CA, USA) was added to the Petri dish. After culturing for 2 days, the fluid was replaced with fresh medium, and the tissue samples were cultured until the cell density reached 90% for passaging.

### Immunofluorescence

GEFs of the third generation were subjected to immunofluorescence staining as previously described (24, 33). Briefly, the GEFs were fixed in 4% paraformaldehyde at 25°C for 20 min, permeabilized with 0.1% Triton X-100 for 30 min, and subsequently blocked for 30 min with 5% bovine serum albumin at 25°C. The GEFs were then incubated with anti-vimentin (a marker of stromal cells, Abcam, Cambridge, MA, USA, ab8978, 1:100), anti-cytokeratin (a marker of epithelial cells, Abcam, ab181597, 1:100), anti-BMAL1 (Abcam, ab93806, 1:100), or anti-NR1D1 (Abcam, ab174309, 1:100) antibodies diluted in PBS at 4°C for 24 h. After washing, the cells were treated with PBS containing fluorescein-conjugated secondary antibody (1:200, Thermo Fisher, Shanghai, China) and DAPI (blue), and the immunostaining signal was detected using a fluorescence microscope (Nikon, Tokyo, Japan).

### RNA Extraction, Reverse Transcription PCR, and Real Time Quantitative PCR

GEFs were synchronized for 2 h with 100 nM dexamethasone (DXM, Sigma-Aldrich, St. Louis, MO, USA) in serum-free DMEM/F-12 medium containing 1 × AA. Cell samples were harvested at the indicated time points (after DXM synchronization for 24, 28, 32, 36, 40, or 44 h). Total RNA was extracted and purified from GEFs and goat liver and kidney tissues using TRIzol reagent (TaKaRa, Dalian, China) and then treated with RNase-free DNase (TianGen, Beijing, China). cDNA was generated using a PrimeScript RT Reagent Kit (TaKaRa). The primer sets used for the qPCR are listed in Table 1. The qPCR reactions were carried out in a 20 μL reaction solution comprising 10 ng cDNA with specific primers (200 nM) using the Thunderbird SYBR qPCR Mix (Toyobo, Osaka, Japan) and a CFX96™ qRT-PCR system (Bio-Rad, Hercules, CA, USA), as described previously (34, 35). All reactions were performed in triplicate. The relative expression levels of each sample were normalized to the average level of the constitutively expressed housekeeping gene, *RPLP0* (also known as *36B4*).

**Table 1:** Primer sequences for the goat targeted genes in qPCR.

**Gene**	**Accession no**.	**Sequence (5^**′**^-3^**′**^)**	**Amplicon (bp)**
*BMAL1*	XM_018059578.1	F: AAGCTTCTGCACAATCCACA R: AAATAGCTGTTGCCCTCTGGT	251
*CLOCK*	XM_018049466.1	F: AGGGGTCACTGACTACGCAT R: GTGTTTTTCTTTCACGGGAGC	148
*NPAS2*	XM_018054924.1	F: TCTGTGACATTCAGCCGGAC R: GATCCATGACATCCGACGGT	177
*DBP*	XM_018062728.1	F: TGGCCATGAGACCTTTGACC R: ATTCTTGTACCGTCGGCTCC	142
*NR1D1*	XM_018065020.1	F: CCCTGGCTTCCGTGATCTTT R: CCTTCACGTTGAACAGCGAC	104
*RORα*	NM_001285652.1	F: CGCAGCGATGAAAGCTCAAA R: AGGACAGGAGTAGGTGGCAT	148
*PER1*	XM_018064610.1	F: CAGGATGTGGATGAGAGGGC R: TTACGGGCACAGAAGCGAAT	182
*PER2*	XM_018040197.1	F: TTCCAGGACGTGGACCAA R: CCCCAGACTGCACGATCTTC	142
*PER3*	XM_005690741.3	F: CTCGGGTTACCAAGCTCCTC R: TGCTAGGATGGATGTCCCGA	151
*CRY1*	XM_013964057.2	F: CTGGTCTTGGCAGTGGGAAA R: CAAAGCTCTCCTCCTTACCTAAT	104
*CRY2*	XM_018059193.1	F: TGGGGACTACATCAGGCGATA R: GGGTAGTCCACGCCAATGAT	126
*E4BP4*	XM_018052510.1	F: GAGCAGGATCACGGTCATCTA R: CTGCATCAGCTGCACCTTAAC	194
*36B4*	NM_001314331.1	F: GGGCAAGAACACGATGATGC R: CCACATTTCCCCGGATGTGA	95

### Protein Extraction and Western Blotting

Total proteins were extracted from the GEF, goat liver, and kidney tissues collected at the indicated time points using a protein extraction kit (Beyotime, Shanghai, China), as previously described (36, 37). The BCA method (Kagan Biotechnology, Nanjing, China) was used to detect total protein concentrations. Total protein was mixed with 5 × loading buffer after boiling for 10 min at 100°C. Equivalent amounts of total protein (20 μg) were separated by 12% sodium dodecyl sulfate-polyacrylamide gel electrophoresis and transferred to a polyvinylidene difluoride membrane (Roche Diagnostics GmbH, Mannheim, Germany) using a wet electrophoresis transfer system (Roche Diagnostics GmbH, Mannheim, Germany). The membranes were sealed at 25°C with TBST containing 10% skim dry milk for 2 h and subsequently incubated with primary antibodies at 4°C overnight. Next, the polyvinylidene difluoride membranes were further incubated with horseradish peroxidase (HRP)-conjugated secondary antibodies at 25°C for 1 h. Both antibodies were diluted in antibody diluent (Beyotime). After washing with TBST four times (5 min each), an ECL Super Sensitive Solution Kit (Advansta, Menlo Park, CA, USA) was used to visualize the protein bands. In addition, the gel imaging system (GBoxChemi-XRQ, Syngene, Cambridge, UK) and Quantity One software (Bio-Rad Laboratories, Hercules, CA, USA) were used to visualize and digitally analyze the immunoreactive bands, respectively. Finally, the relative protein expression levels were normalized to β-actin levels in the samples. The primary antibodies used were rabbit anti-BMAL1 (Abcam, ab93806, 1:3000), rabbit anti-NR1D1 (Abcam, ab174309, 1:3000), and mouse anti-β-actin (Sanjian Biotech, Tianjin, China, 1:5000). The secondary antibodies used were horseradish peroxidase-conjugated antibodies (Zhongshan Golden Bridge Biotechnology, Nanjing, China, Catalog Number: PB001, 1:5000).

### Genes Cloning and Construction of Eukaryotic Expression Vectors

Genomic DNA was extracted from GEFs and NIH3T3 cells, respectively, using a genomic DNA extraction kit (TIANamp Genomic DNA Kit, TianGen). Total RNA and cDNAs of goats were obtained as described above. The primer sequences for the coding sequences (CDS) of goat clock genes (*BMAL1, CLOCK, CRY2, ROR*α, and *NR1D1*) and the promoter regions of goat and mouse clock genes (*BMAL1* and *NR1D1*) were designed using Primer Premier 6.0 bio-software (Premier, Palo Alto, CA, USA). All primer sequences are shown in Table 2. Genomic DNA or cDNA was used as a template for PCR amplification using Primer STAR HS DNA Polymerase (TaKaRa). PCR products were separated by electrophoresis on a 1% agarose gel, followed by purification using a Universal DNA Purification Kit (TianGen). The CDS fragments of goat clock genes (*BMAL1, CLOCK, CRY2, ROR*α, and *NR1D1*) were subcloned into the pcDNA3.1 plasmid using the seamless connection method (ClonExpress II One Step Cloning Kit, Vazyme, Nanjing, China), respectively. The proximal gene promoter regions of goat *BMAL1* (−684 to + 178; the transcription start site is regarded as + 1) and goat *NR1D1* (−1421 to + 236) were subcloned into the pGL4.10 and pGL4.22 vectors (Promega, Madison, WI, USA), respectively, using the seamless connection method (ClonExpress II One Step Cloning Kit, Vazyme). The proximal gene promoter regions of mouse *BMAL1* (−593 to +197) and mouse *NR1D1* (−1475 to −25) were subcloned into the pGL4.22 vectors, respectively, using the seamless connection method.

**Table 2:** PCR primer sequences for clock genes and circadian gene promoters.

**Gene**	**Accession no**.	**Sequence (5^**′**^-3^**′**^)**	**Enzyme restriction site (underlined part)**	**Amplicon (bp)**
*gBMAL1*	XM_018059578.1	F: AGCAAGCTTTCTAGAGAATTCATGGCCGACCAGAGAATGGAC R: ACCGGATCCGATATCGAATTCTTACAGCGGCCATGGCAAGTC	*Eco*RI	1,881
*gCLOCK*	XM_018049467.1	F: ATCGGATCCGGTACCCTCGAGATGTTGTTTACCGTAAGCTGTA R: TTTAAACTTACCGGTCTCGAGCTACTGTGGTTGAACCTTGGAA	*Xho*I	2,538
*gCRY2*	XM_018059193.1	F: AGCAAGCTTTCTAGAGAATTCATGGCGGCGGCGGCAGCGGCGA R: ACCGGATCCGATATCGAATTCTCAGACGCCCCTGCTCGGCAGT	*Eco*RI	1,791
*gRORα*	NM_001285652.1	F: AGCAAGCTTTCTAGAGAATTCATGATGTATTTTGTGATCGCAG R: ACCGGATCCGATATCGAATTCTTACCCATCAATCTGCATGGCT	*Eco*RI	1,407
*gNR1D1*	XM_018065020.1	F: AGAGAATTCGATATCGGATCCATGACGACCCTGGACTCTAACA R: GGTCTCGAGGGTACCGGATCCTCACTGGGCGTCCACCCGGAAG	*Bma*HI	1,851
*gBMAL1-promoter*	NC_030822.1	F: CTGGCCTAACTGGCCGGTACCTGGAGGTCAGGAAGAACGAA R: AGGCTAGCGAGCTCAGGTACCCCGACACTCACCGTGGCTC	*Kpn*I	862
*gNR1D1-promoter*	NC_030826.1	F: CCTGAGCTCGCTAGCCTCGAGTTCAGTCACCATCACAGTC R: CCAGATCTTGATATCCTCGAGGCAGTCAGGAAGTAAGTAGG	*Xho*I	1,657
*mBMAL1-promoter*	NC_000073.7	F: CTGGCCTAACTGGCCGGTACCGGTTCCGACTAGGACAAGC R: AGGCTAGCGAGCTCAGGTACCGCCATGCCGACACTCA	*Kpn*I	790
*mNR1D1-promoter*	NC_000077.7	F: CCTGAGCTCGCTAGCCTCGAGACGAGGGAACAGGGCTAA R: CCAGATCTTGATATCCTCGAGGGATTGTTGGGATTTGTAGTC	*Xho*I	1,451

### Bioinformatics Analysis

The amino acid sequences of BMAL1, CLOCK, CRY2, RORα, and NR1D1 from goats, cows, mice, and humans were obtained from the National Center for Biotechnology Information (NCBI) database. The Basic Local Alignment Search Tool (https://blast.ncbi.nlm.nih.gov/Blast.cgi) on the NCBI website was used to compare the similarities between amino acid sequences of circadian clock proteins from different species. SWISS-MODEL (https://swissmodel.expasy.org/interactive) was used to predict the tertiary structures of the BMAL1 and CLOCK proteins in goats, mice, and humans. The ZDOCK SERVER (https://zdock.umassmed.edu/) program was used to predict the tertiary structure of the BMAL1/CLOCK heterodimer in goats, mice, and humans. Finally, differences in the tertiary structures of the circadian clock proteins were compared using PyMOL 2.3.2 software (Schrodinger LLC, Cambridge, UK).

### Dual-Luciferase Reporter Assay

HEK293T cells were provided by the Chinese Academy of Sciences Cell Bank (Shanghai, China) and grown in DMEM supplemented with 10% FBS (Gibco) and 1 × AA (Invitrogen) in a humidified atmosphere with 5% CO_2_ at 37°C. The relevant plasmids, including 100 ng of promoter reporters (pGL4.10-gBMAL1-Promoter-*Luc* (pGL4.10-gBMAL1-P-*Luc*) or pGL4.10-gNR1D1-Promoter-*Luc* (pGL4.10-gNR1D1-P-*Luc*)) plasmid, 5 ng of pRL-CMV (Promega, an internal control plasmid), and different doses of expression vectors (depending on the experiment) were co-transfected into HEK293T cells grown in 24-well plates using Turbofect transfection reagent (Thermo Fisher Scientific, Vilnius, Lithuania). The total DNA amount was made equal among all conditions by the addition of pcDNA3.1 empty vector whenever required. After 36 h post-transfection, cell lysis buffer (100 μL per sample) was used to lyse the cells, and the relative luciferase activity was measured using the Dual-Luciferase Reporter Assay System (Promega) and SPARK ^®^ Multimode Microplate Reader (Tecan, Männedorf, Switzerland), as described previously (38). Renilla luciferase was used as a control.

### Real-Time Monitoring of Luminescence

NIH3T3 cells were provided by the Chinese Academy of Sciences Cell Bank (Shanghai, China) and grown in DMEM supplemented with 10% FBS (Gibco) and 1 × AA (Invitrogen). GEFs were grown in DMEM/F12 supplemented with 10% FBS (Gibco) and 1 × AA (Invitrogen). Both NIH3T3 cells and GEFs were cultured in a humidified atmosphere containing 95% air and 5% CO_2_ at 37°C. The cells were plated in 35-mm dishes at a density of 1.5 × 10^5^ cells/dish and incubated for 24 h before transfection with promoter reporters (pGL4.22-gBMAL1-promoter-*Luc* or pGL4.22-gNR1D1-promoter*-Luc* or pGL4.22-mBMAL1-promoter-*Luc* or pGL4.22-mNR1D1-promoter*-Luc* plasmids) using Turbofect transfection reagent (Thermo Fisher Scientific). After transfection for 24 h, the cells were synchronized for 2 h with 100 nM DXM in serum-free DMEM or DMEM/F12. The culture medium was then changed to DMEM or DMEM/F12 supplemented with 100 μM luciferin (GoldBio, St. Louis, MO, USA) and 1 × AA (Invitrogen) for luminescence determination. Luciferase activity was chronologically monitored at 37°C with a Kronos Dio AB-2550 luminometer (ATTO, Tokyo, Japan) interfaced with a computer for continuous data acquisition, as described previously (39, 40). The amplitudes and periods of the goat *BMAL1* and *NR1D1* promoters driving luciferase oscillations were analyzed and obtained using the single Cosinor method (41).

### Statistical Analyses

All data are expressed as the mean ± standard error (SE) of at least three independent experiments, each performed in triplicate. The circadian rhythmicity of gene expression was detected by the single Cosinor method using Time Series Single 6.3 software (Expert Soft Tech, Richelieu, France). Rhythmic expression was defined as *P-*value < 0.05. Statistical analysis was conducted using Student's *t*-test and one-way analysis of variance (ANOVA) with Tukey's multiple comparisons post-test using GraphPad Prism version 7.0 (GraphPad, San Diego, CA, USA). Differences were considered statistically significant at *P* < 0.05.

## Results

### Isolation and Identification of GEFs

**Figure 1:**
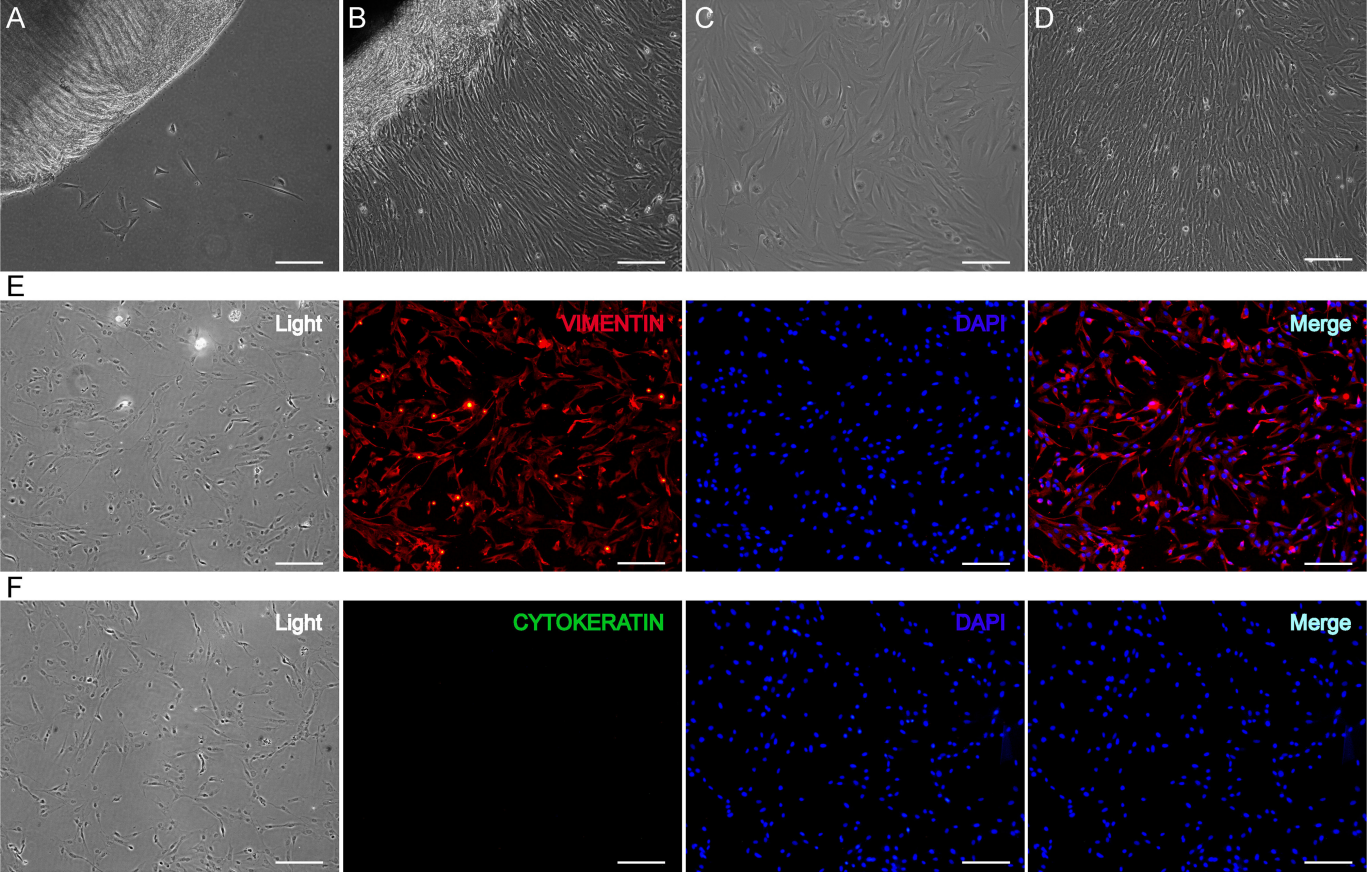
Isolation and identification of GEFs. GEFs were isolated from 30-day gestational goat fetuses by tissue block attachment method. The migration of the cells was observed under an inverted microscope after 3 days **(A)** and 9 days **(B)** of adhesion. The cells that migrated from the tissue blocks were digested and collected, and the cell suspensions were re-inoculated into new petri dishes. The cell status was observed under an inverted microscope on the 2nd day **(C)** and 4th day **(D)** after subculture. Third-generation GEFs were used for cell immunofluorescence staining. After culture for 2 days, the attached GEFs were subjected to fluorescent immunocytochemistry using anti-vimentin (red, **E**) and anti-cytokeratin (green, **F**) antibodies. Scale bar, 50 μm. GEFs, goat embryonic fibroblasts.

GEFs were isolated from 30-day gestational goat fetuses using the tissue block attachment method. After 3 days of adherent culture, a few cells migrated from the edge of the tissue block (Figure 1A). After 9 days of adherent culture, the cells grew and reached a density of > 90% (Figure 1B). After subculturing, the cells displayed a long spindle shape and good growth state (Figures 1C,D). Third-generation GEFs were used for immunofluorescence staining. Immunofluorescence staining was performed using vimentin (a characteristic marker of fibroblasts) and cytokeratin (a characteristic marker of epithelial cells), respectively (42). The results showed positive vimentin staining (Figure 1E) and negative cytokeratin staining (Figure 1F). These results showed that the obtained cells were GEFs with high purity, providing an ideal cell model for follow-up research.

### MRNA Expression Profiles of Clock Genes and Clock-Controlled Genes in GEFs Over 20 h Following DXM Stimulation

**Figure 2:**
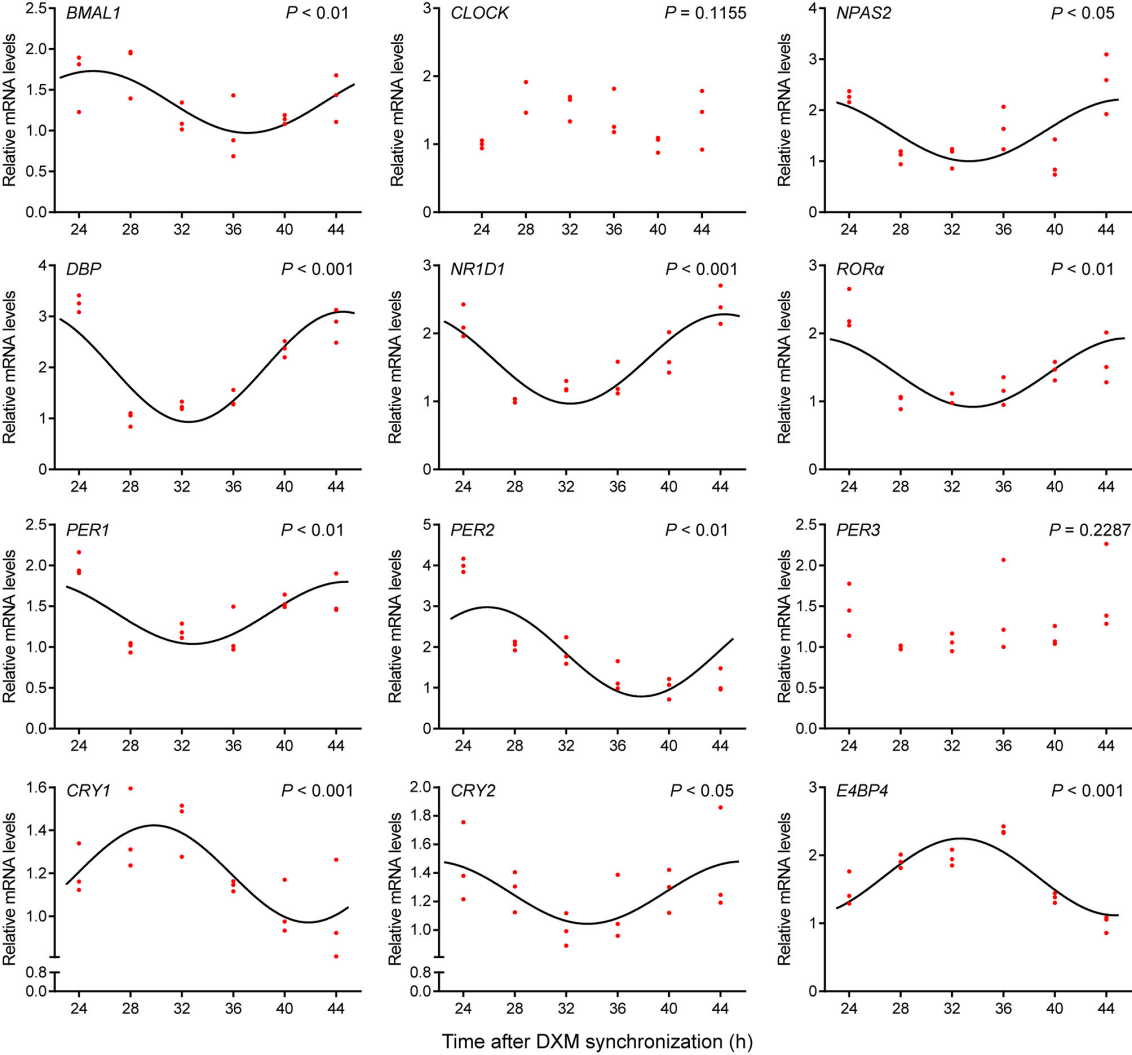
Expression profiles of clock genes in GEFs. Total RNA samples were collected at the indicated times following DXM synchronization. The mRNA expression levels of the clock genes (*BMAL1, CLOCK, NPAS2, NR1D1, ROR*α, *PER1, PER2, PER3, CRY1*, and *CRY2*) and clock-controlled genes (*DBP* and *E4BP4*) were normalized to the housekeeping gene *36B4* and expressed as relative to the nadir. Red dots represent the relative values of each independent determination at the respective time points (*n* = 3). Cosinor analysis was utilized to determine the rhythmic expression patterns of the examined genes. The black solid lines show the fitted curves. *P* < 0.05 was considered to indicate rhythmic expression. GEFs, goat embryonic fibroblasts.

To investigate the circadian clock system in GEFs, cells were synchronized with 100 nM DXM for 2 h, after which the medium was changed. Cell samples were collected at 24, 28, 32, 36, 40, and 44 h, following medium change. The mRNA expression patterns of several canonical clock genes and clock-controlled genes were examined using qPCR. As shown in Figure 2, the *BMAL1* mRNA expression levels exhibited significant circadian variations (Cosinor analysis, *P* < 0.01), reaching their lowest expression levels at 37.1 h after DXM synchronization. The mRNA expression levels of other clock genes (*NPAS2, NR1D1, ROR*α, *PER1*, and *PER2*) and clock-controlled genes (*DBP* and *E4BP4*) also displayed rhythmic circadian changes with double-to-quadruple-fold changes (peak to nadir) over the 20-h sampling period (Figure 2, Cosinor analysis, *P* < 0.05). In addition, *CRY1* and *CRY2* mRNA expression profiles also showed weak but rhythmic changes (Figure 2, Cosinor analysis, *P* < 0.001 and *P* < 0.05, respectively), while *CLOCK* and *PER3* transcripts were expressed at constant levels (Figure 2, Cosinor analysis, *P* = 0.1155 and *P* = 0.2287, respectively). Notably, all of the transcript levels of *NPAS2, DBP, NR1D1, ROR*α, *PER1*, and *CRY2* were out of phase with those of *BMAL1*. These data indicate the existence of a functional circadian clock system in the GEFs.

### Circadian Variations of BMAL1 and NR1D1 Protein Levels Are Present in GEFs Following DXM Stimulation

**Figure 3:**
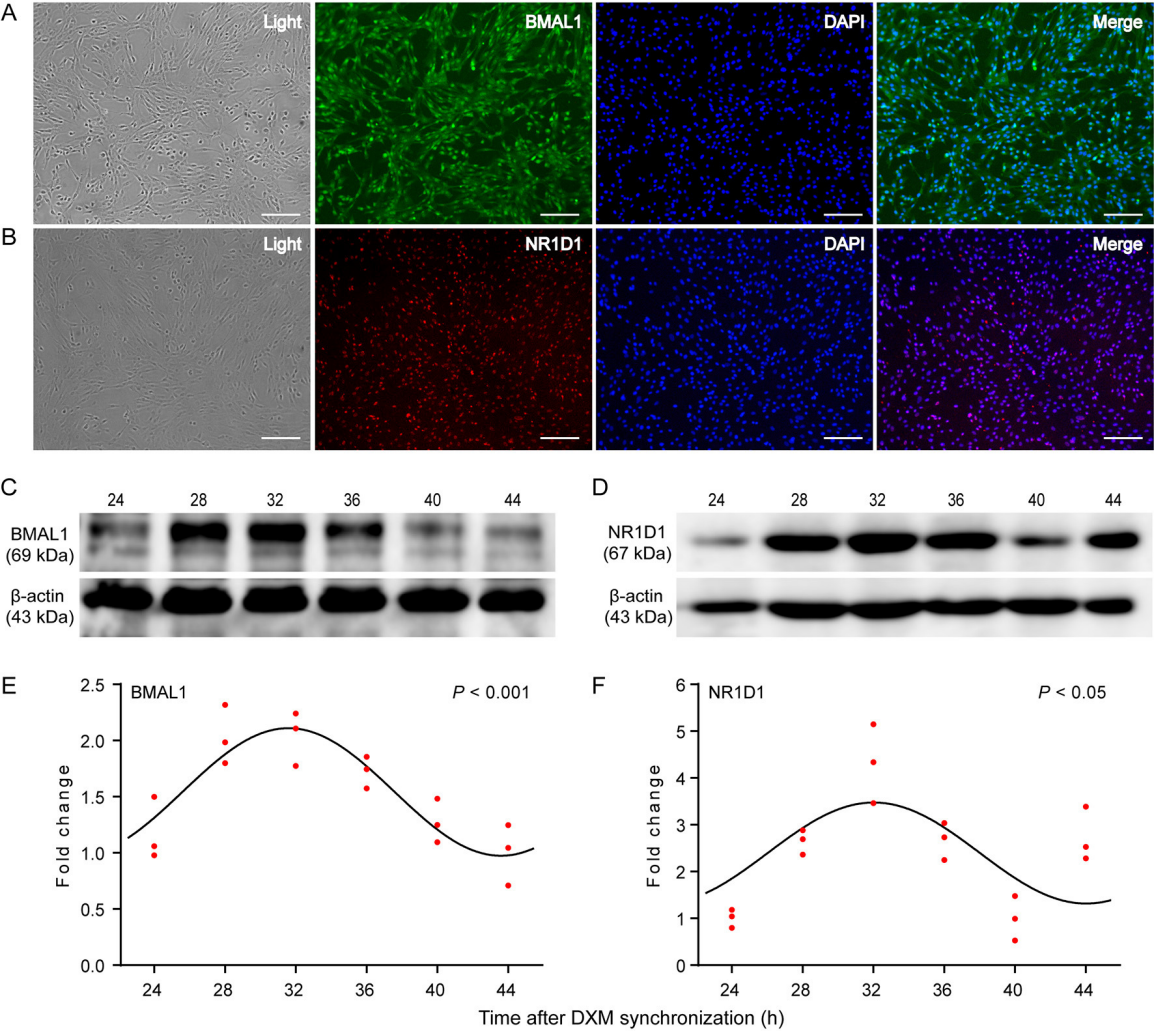
Expression patterns of the circadian clock proteins in GEFs. Third-generation GEFs were used for cell immunofluorescence staining. After culture for 2 days, the attached GEFs were subjected to fluorescent immunocytochemistry using anti-BMAL1 (green, **A**) and anti-NR1D1 (red, **B**) antibodies. Scale bar, 50 μm. Total proteins were extracted from the GEFs and collected at the indicated times following DXM synchronization. The protein levels of BMAL1 **(C)** and NR1D1 **(D)** were analyzed by western blotting analysis. The ratio of BMAL1 **(E)** or NR1D1 **(F)** protein to β-actin was determined from the densities of the immunoreactive bands and expressed as relative to the nadir. Red dots represent the relative values of each independent determination at the respective time points (*n* = 3). Cosinor analysis was utilized to determine the rhythmic expression patterns of the examined proteins. The black solid lines show the fitted curves. *P* < 0.05 was considered to indicate rhythmic expression. GEFs, goat embryonic fibroblasts.

BMAL1 and NR1D1, two core clock components, were examined to determine their distribution and expression patterns in GEFs using immunofluorescence staining and WB. As shown in Figures 3A,B, the endogenous expression levels of BMAL1 and NR1D1 were detected. More specifically, BMAL1 was expressed in both the nucleus and cytoplasm of GEFs (Figure 3A), whereas NR1D1 expression was highly distributed in the nucleus (Figure 3B). GEF samples were collected at 24, 28, 32, 36, 40, and 44 h for WB analysis, following DXM synchronization. BMAL1 and NR1D1 protein levels exhibited significant circadian changes over a 20-h sampling period (Figures 3C–F, Cosinor analysis, *P* < 0.001 and *P* < 0.05, respectively). In summary, these data further support the existence of a functional circadian clock system in the GEFs.

### Diurnal Changes of Clock Genes Expression Are Present in Male Goat Liver and Kidney Tissues

**Figure 4:**
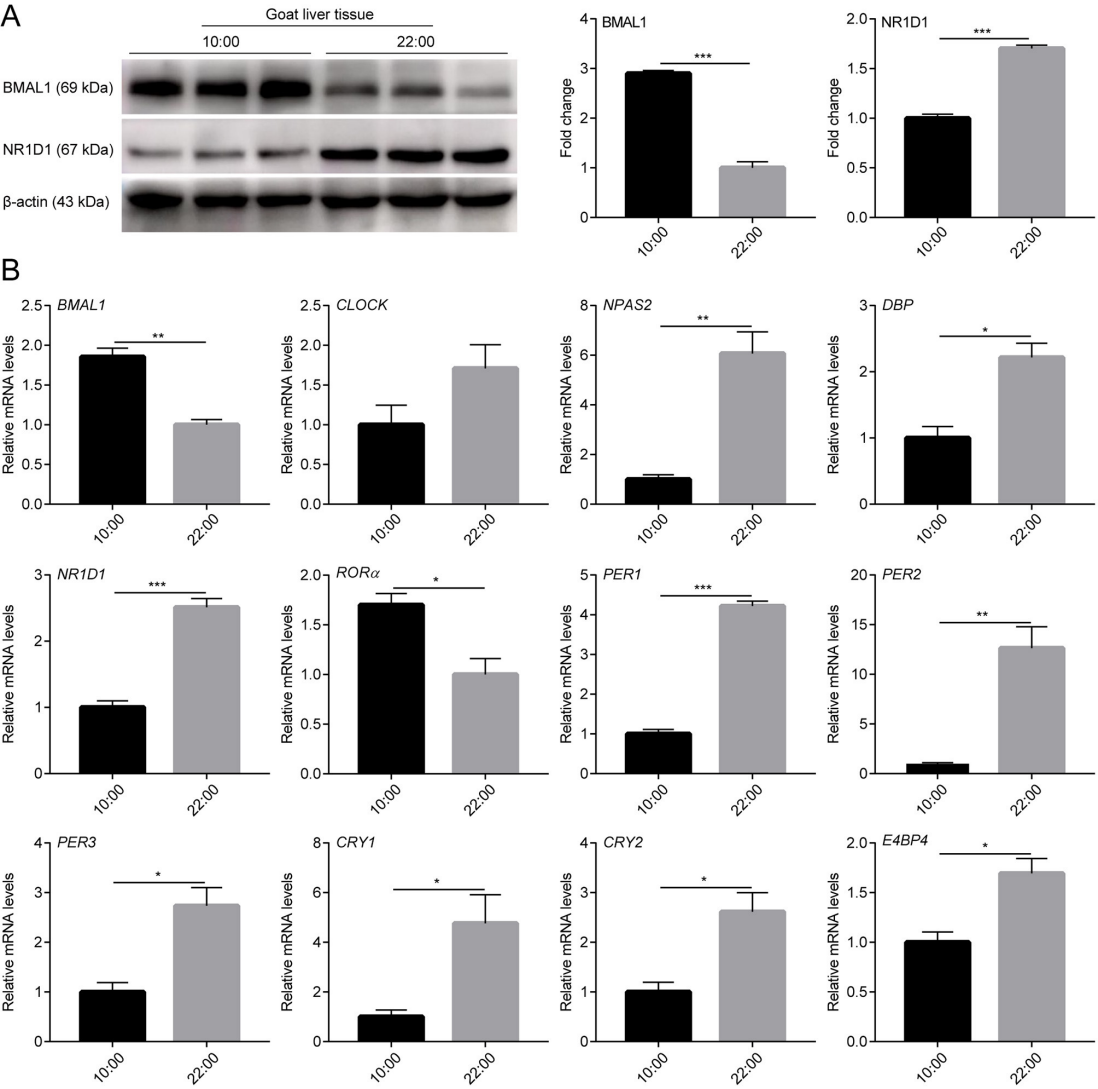
mRNA and protein expression profiles of clock genes in male goat liver tissues. The male goat liver tissues were collected at two representative time points (10:00 h and 22:00 h). **(A)** The expression levels of BMAL1 and NR1D1 were examined by western blotting analysis. Representative blots and quantitative analysis from three independent experiments are shown. **(B)** The mRNA expression levels of clock genes (*BMAL1, CLOCK, NPAS2, NR1D1, ROR*α, *PER1, PER2, PER3, CRY1*, and *CRY2*) and clock-controlled genes (*DBP* and *E4BP4*) were examined by qPCR. All data are presented as means ± SE (*n* = 3). Student's *t*-test was used to analyze the difference within different groups. Differences were considered significant at *P* < 0.05. Asterisks indicate significant differences. *P < 0.05, **P < 0.01, ***P < 0.001.

**Figure 5:**
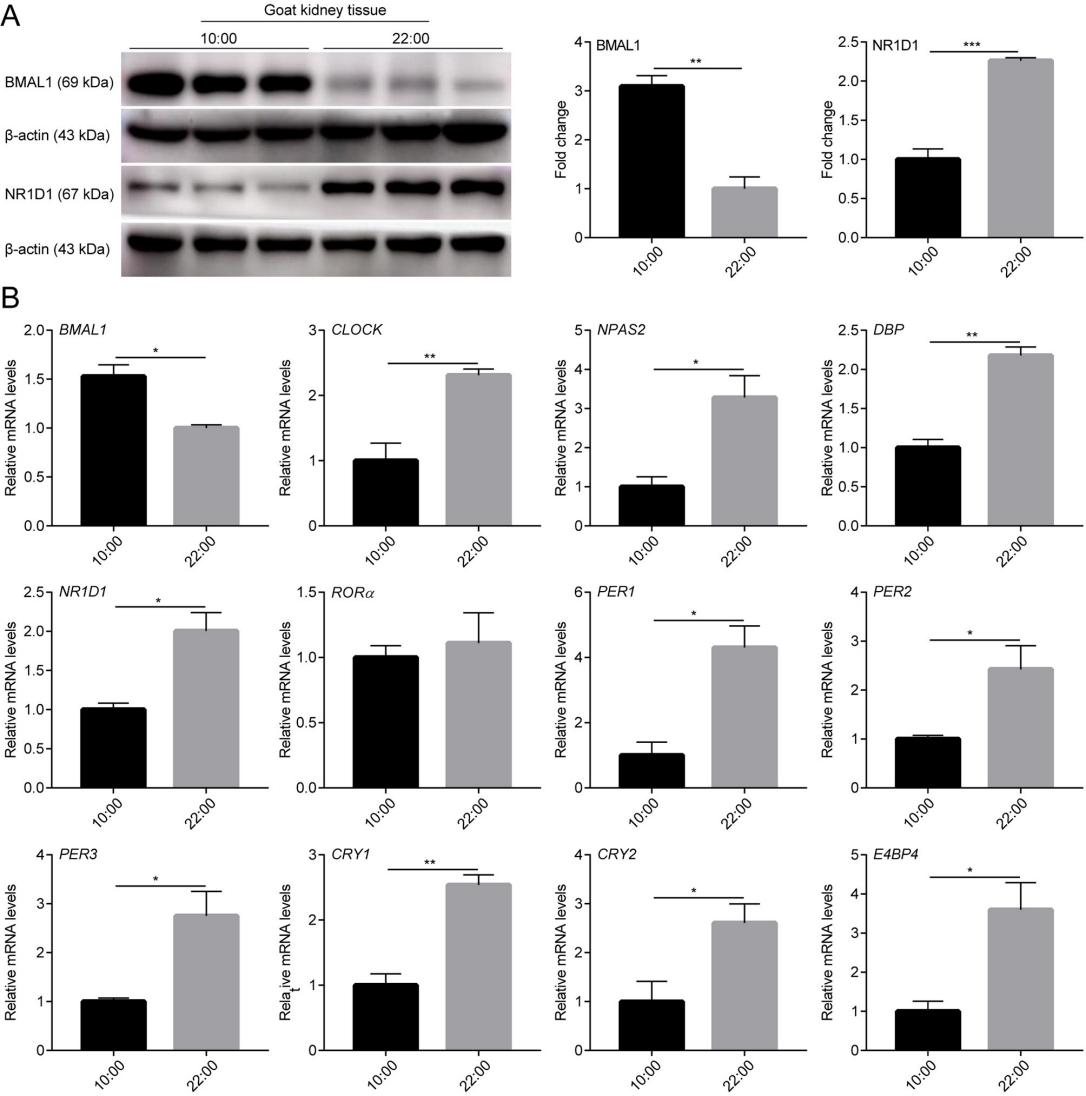
mRNA and protein expression profiles of clock genes in male goat kidney tissues. The male goat kidney tissues were collected at two representative time points (10:00 h and 22:00 h). **(A)** The expression levels of BMAL1 and NR1D1 were examined by western blotting analysis. Representative blots and quantitative analysis from three independent experiments are shown. **(B)** The mRNA expression levels of clock genes (*BMAL1, CLOCK, NPAS2, NR1D1, ROR*α, *PER1, PER2, PER3, CRY1*, and *CRY2*) and clock-controlled genes (*DBP* and *E4BP4*) were examined by qPCR. All data are presented as means ± SE (*n* = 3). Student's *t*-test was used to analyze the difference within different groups. Differences were considered significant at *P* < 0.05. Asterisks indicate significant differences. *P < 0.05, **P < 0.01, ***P < 0.001.

To determine whether there were rhythmic changes in clock genes expression in goats *in vivo*, the expression profiles of several clock genes (*BMAL1, CLOCK, NPAS2, NR1D1, ROR*α, *PER1, PER2, PER3, CRY1*, and *CRY2*) and clock-controlled genes (*DBP* and *E4BP4*) in male goat liver and kidney tissues collected at two representative time points (10:00 and 22:00) were examined using a qPCR assay. As shown in Figures 4B, 5B, the majority of clock genes (*BMAL1, NPAS2, NR1D1, PER1, PER2, PER3, CRY1*, and *CRY2*) and clock-controlled genes (*DBP* and *E4BP4*) exhibited robust diurnal changes in their mRNA abundance in male goat liver and kidney tissues. Notably, *NPAS2, DBP, NR1D1, PER1, PER2, PER3, CRY1, CRY2*, and *E4BP4* exhibited anti-phase expression patterns with respect to *BMAL1* in both the liver and kidney tissues (Figures 4B, 5B). It should be mentioned that *CLOCK* exhibited a constant expression level in liver tissues, whereas significant changes were detected in kidney tissues (Figures 4B, 5B). In contrast, *ROR*α exhibited a constant expression level in kidney tissues, whereas significant changes were detected in the liver tissues (Figures 4B, 5B).

In addition, BMAL1 and NR1D1 protein levels were detected in the liver and kidney tissues of male goats at the two representative time points using WB. As shown in Figures 4A, 5A, BMAL1 and NR1D1 protein levels exhibited significant diurnal changes in the liver and kidney tissues of male goats. As expected, NR1D1 displayed an anti-phase expression pattern with respect to BMAL1 (Figures 4A, 5A), which was consistent with the changes in their mRNA expression profiles. These results indicate that there is a functional circadian clock system in goat liver and kidney tissues.

### Cloning of the Promoter Regions and CDS Fragments of Clock Genes and Their Bioinformatics Analysis

**Figure 6:**
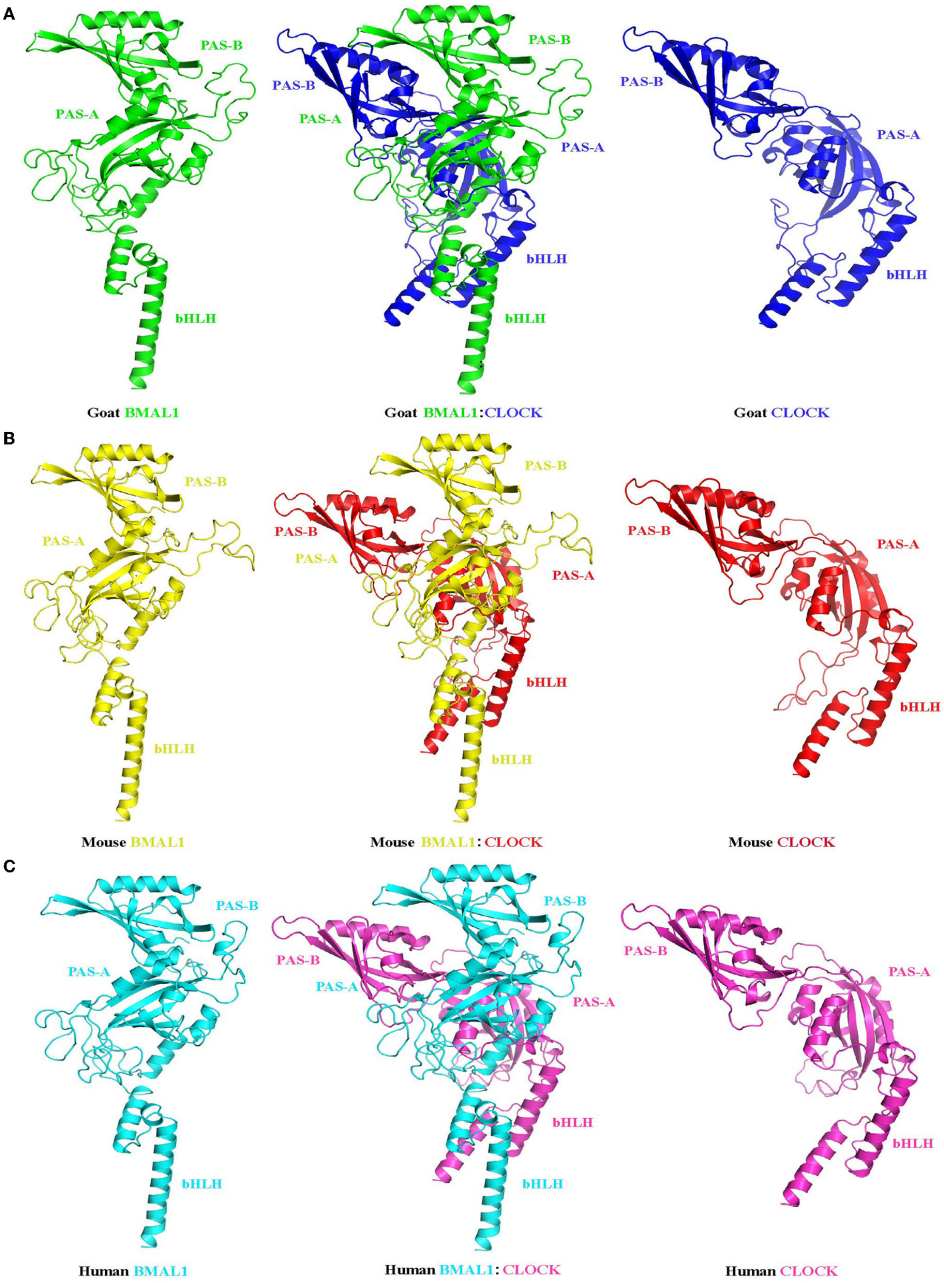
Prediction of the tertiary structure of the circadian clock core transcription factors BMAL1 and CLOCK and their heterodimers in goats **(A)**, mice **(B)**, and humans **(C)**. Ribbon diagram of BMAL1: CLOCK heterodimer (center). Each individual domain is labeled. The BMAL1 (left) and CLOCK (right) subunits are also shown separately to illustrate their different spatial domain arrangements. PAS-A: Per-Arnt-Sim A domain; PAS-B: Per-Arnt-Sim B domain; bHLH: basic helix-loop-helix domain.

Although mRNA and protein expression levels of clock genes have been shown to exhibit circadian changes in goats, the underlying molecular regulatory mechanisms remain unclear. To examine whether the transcriptional and translational feedback loops of the goat circadian clock system were conserved in mammals, the CDS (*BMAL1, CLOCK, CRY2, ROR*α, and *NR1D1*) and promoter region (*BMAL1* and *NR1D1*) fragments of goat clock genes were cloned. Table 3 provides detailed information about the cloned goat clock genes, including their size, GenBank accession numbers, and protein identities, compared to their orthologs in cows, mice, and humans, when applicable. Full-length CDS fragments of several goat clock genes, including the transcriptional activators BMAL1 and CLOCK, the transcriptional repressor CRY2, and two members of the nuclear receptor family, namely NR1D1 and RORα, were obtained (data not shown). The homology of the amino acid sequences of these genes across mammals (goat, cow, mouse, and human) is very high, with percentages above 93%. The tertiary structures of BMAL1, CLOCK, and their heterodimers in goats, mice, and humans were analyzed and predicted (Figure 6). To compare the similarity of BMAL1/CLOCK heterodimers in different species, the root mean squared deviation (RMSD) values between their pairwise structures were analyzed. RMSD is a numerical measure of the difference between two structures, with a smaller RMSD value denoting more similarity between them (43). The RMSD value of the goat to mouse BMAL1/CLOCK protein complex was 3.416, that of goat to human was 2.824, and that of human to mouse was 0.933. This indicates that the tertiary structures of BMAL1/CLOCK proteins in goats, mice, and humans were highly similar, and there was higher homology between goats and humans than between mice and goats.

**Table 3:** Cloning of a comprehensive set of clock genes and circadian gene promoters in the goat.

	**Size (bp)**	**GenBank accession**	**Protein identity (%)**		
			**Cow**	**Mouse**	**Human**
**Expression vectors**					
*BMAL1*	1881	XM_018059578.1	99	96	98
*CLOCK*	2538	XM_018049467.1	99	93	97
*CRY2*	1791	XM_018059193.1	93	93	95
*RORα*	1407	NM_001285652.1	99	99	98
*NR1D1*	1851	XM_018065020.1	99	93	94
**Gene promoter reporters**
*BMAL1*	862	NC_030822.1			
*NR1D1*	1657	NC_030826.1			

*The complete coding sequence of five clock genes was cloned and used to construct the expression vectors. Proximal gene promoter regions of the Capra hircus BMAL1 and NR1D1 were also obtained. The table provides the size, GenBank accession number, and protein homology when applicable*.

**Figure 7:**
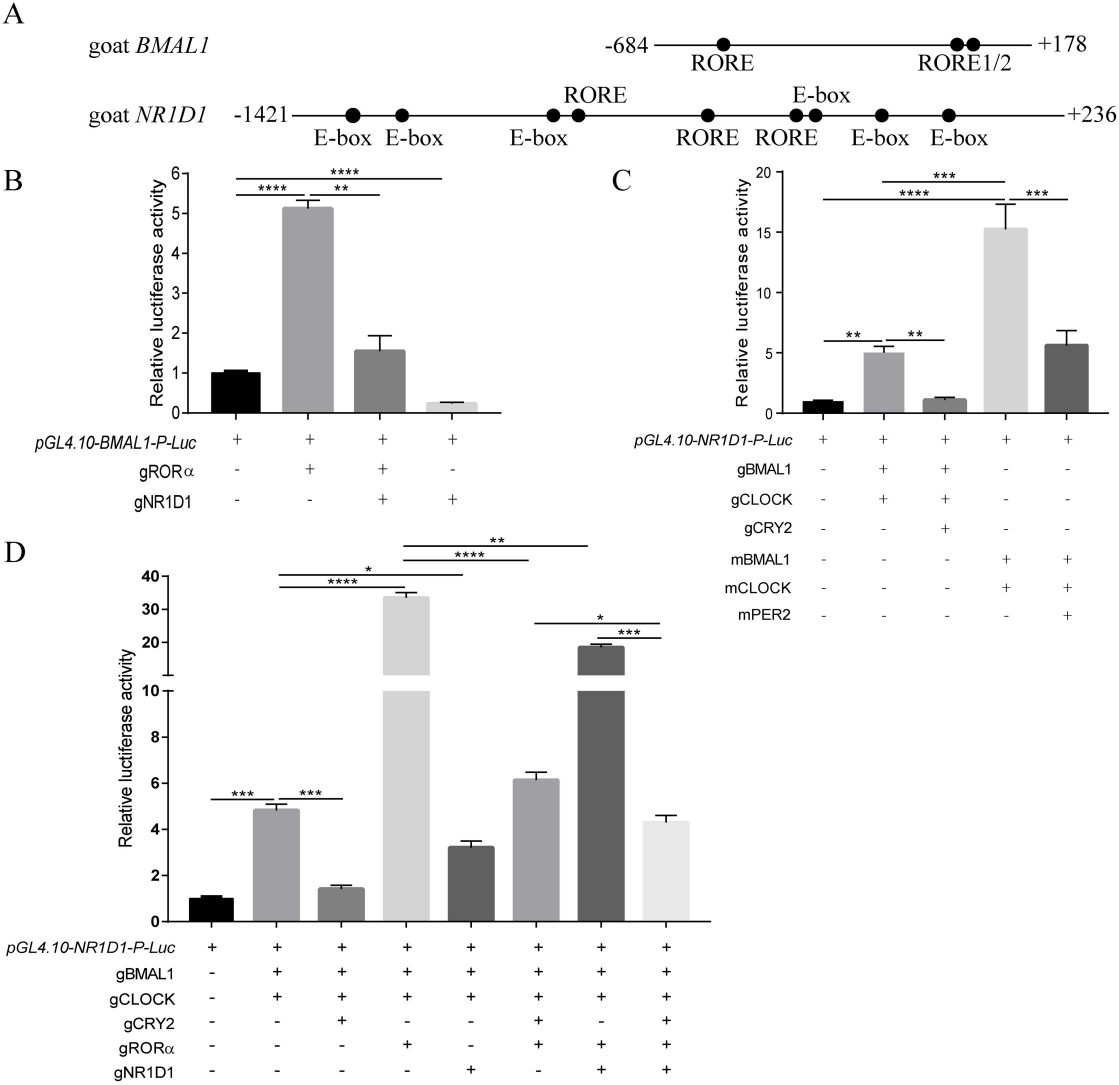
Transcriptional activity of the goat *BMAL1* and *NR1D1* gene *in vitro*. Schematics of the *BMAL1* and *NR1D1* promoter regions in goat **(A)**. Positions relative to the goat transcriptional start site are indicated. Dots indicate response elements. The roles of transcriptional activators (gRORα, gBMAL1/gCLOCK, mBMAL1/mCLOCK) and repressors (gNR1D1, gCRY2, mPER2) in the caprine circadian clock was investigated by luciferase assays in HEK293T cells. Transcriptional activation of the luciferase reporter containing an 862 bp fragment from the 5′-flanking region of goat *BMAL1* **(B)**. Transcriptional activation of the luciferase reporter containing a 1657 bp fragment from the 5′-flanking region of goat *NR1D1* **(C,D)**. Data are presented as the mean ± SE (*n* = 3) of firefly luciferase activity (pGL4.10-gBMAL1-P-*Luc* or pGL4.10-gNR1D1-P-*Luc*) normalized with the Renilla luciferase control (pRL-CMV) from a single assay. The results shown are representative of three independent experiments. The activity in samples transfected with the reporter construct (i.e., pGL4.10-gBMAL1-P-*Luc* or pGL4.10-gNR1D1-P-*Luc* without plasmids expressing other proteins) was set to 1.0. *P*-values were calculated using one-way ANOVA with Tukey's multiple comparison post-test. Asterisks indicate significant differences. *P < 0.05, **P <0.01, ***P < 0.001, ****P < 0.0001.

Table 4 lists a summary of the specific information of putative clock-controlled elements at the promoters of goat *BMAL1* and *NR1D1*, including gene accession numbers, element names, element sequences, chromosomal positions, and gene positions. Genomic inspection revealed that there are three putative RORE elements in both 862 bp of the 5′-flanking region in goat *BMAL1* and 1657 bp of the 5′-flanking region in goat *NR1D1* (Figure 7A). In addition, six E-box motifs were found within 1657 bp of the 5′-flanking region in goat *NR1D1* (Figure 7A). Therefore, the promoter regions of goat *NR1D1*, which contains a hybrid promoter, and goat *BMAL1* were cloned (Table 3) and used to construct a luciferase reporter vector (data not shown).

**Table 4:** Location of putative clock-controlled elements at the promoters of goat genes investigated in the present study.

**Gene**	**Accession No**.	**Elements**	**Sequence 5^**′**^-3^**′**^**	**Chromosomal position** **(Assembly: ASM170441v1)**	**Gene position** **(Transcription start site is +1)**
*BMAL1*	NC_030822.1	RORE	TGACCCAGAA	chr 15 (+) 42932338 to 42932347	−451 to −442
		RORE	AAGTAGGTTA	chr 15 (+) 42932814 to 42932823	+25 to +34
		RORE	AAGTAGGTCA	chr 15 (+) 42932850 to 42932859	+61 to +70
*NR1D1*	NC_030826.1	E-box	CAAGTG	chr 19 (–) 40046708 to 40046703	−1273 to −1268
		E-box	CAGGTG	chr 19 (–) 40046621 to 40046616	−1186 to −1181
		E-box	CAAGTG	chr 19 (–) 40046286 to 40046281	−851 to −846
		RORE	TGACCTTGGG	chr 19 (–) 40046262 to 40046253	−827 to −818
		RORE	AAAGGGGTCA	chr 19 (–) 40045905 to 40045896	−470 to −461
		RORE	GACAGGGTCA	chr 19 (–) 40045789 to 40045780	−354 to −345
		E-box	CACGTG	chr 19 (–) 40045769 to 40045764	−334 to −329
		E-box	CACATG	chr 19 (–) 40045672 to 40045667	−237 to −232
		E-box	CACATG	chr 19 (–) 40045416 to 40045411	+19 to +24

### Characteristic Features of Transcriptional Feedback Loops in Goat Circadian Clock System

To examine whether the transcriptional regulatory mechanism of *BMAL1* in goats was consistent with that in other mammals such as mice, the *BMAL1-luciferase (Luc*) reporter vector containing an 862 bp (−684 to + 178) promoter region fragment for goat *BMAL1* was constructed and subcloned into a pGL4.10[*Luc2*] vector. A dual-luciferase reporter assay was used to detect goat *BMAL1* promoter activity in the presence or absence of gRORα and gNR1D1. As shown in Figure 7B, transfection with gRORα increased pGL4.10-gBMAL1-Promoter-driven luciferase activity (>5-fold changes) compared to pGL4.10-gBMAL1-P*-Luc* plasmid transfection only (control group). In contrast, gNR1D1 severely inhibited the gRORα-induced transcriptional activity (Figure 7B). Notably, the transcriptional activity of the gBMAL1 promoter was significantly inhibited following gNR1D1 overexpression compared with that in the control group (Figure 7B).

In addition, the *NR1D1-luciferase* (*Luc*) reporter vector containing a 1657 bp (−1421 to +236) promoter region fragment for goat *NR1D1* was also constructed and subcloned into a pGL4.10[*Luc2*] vector. The dual-luciferase reporter assay was used to compare the transcription-induction ability of gBMAL1/gCLOCK to that of mBMAL1/mCLOCK toward the promoters described above (Figure 7C). As expected, co-transfection with mBMAL1/mCLOCK and gBMAL1/gCLOCK significantly increased pGL4.10-gNR1D1-Promoter-driven luciferase activity compared to pGL4.10-gNR1D1-P*-Luc* plasmid transfection only (control group). Notably, the increase in pGL4.10-gNR1D1-Promoter-driven luciferase activity induced by the mouse BMAL1/CLOCK overexpression group (compared to the control group, >15-fold change, Figure 7C) was much higher than that in the goat BMAL1/CLOCK overexpression group (compared to the control group, > 5-fold change, Figure 7C). As expected, co-transfection with mPER2 or gCRY2 significantly inhibited mBMAL1/mCLOCK and gBMAL1/gCLOCK transcriptional activity, respectively (Figure 7C).

The goat *NR1D1* promoter is a hybrid promoter that contains both E-Box and RORE motifs (Figure 7A and Table 4). To further decipher the upstream molecular mechanisms regulating *NR1D1* transcription, a dual-luciferase reporter assay was performed on goat *NR1D1* promoter-driven luciferase vectors. As shown in Figure 7D, gRORα and gNR1D1 displayed active and repressive functions, respectively, toward the goat *NR1D1* promoter. When gRORα was co-transfected with gBMAL1 and gCLOCK, the effect on transactivation was additive, and luciferase activity was six times higher than that of the co-transfection of gBMAL1 and gCLOCK groups (Figure 7D). The results also showed that luciferase activity was significantly inhibited when gCRY2 or gNR1D1 was added separately compared with groups without gCRY2 or gNR1D1 overexpression. Furthermore, when the transcriptional repressors gCRY2 and gNR1D1 were added simultaneously, the transcriptional inhibition effect was additive, and luciferase activity was significantly decreased compared with that when gCRY2 or gNR1D1 were added separately (Figure 7D). The above data showed that the main characteristics of the mammalian circadian clock (transcriptional controls and repression by CRY2 and NR1D1) were faithfully recapitulated using caprine clock components, while the activation ability of gBMAL1/gCLOCK was not as robust as that of mBMAL1/mCLOCK.

### Real-Time Monitoring of *BMAL1-Luc* and *NR1D1-Luc* Oscillations in NIH3T3 Cells and GEFs After DXM Synchronization

**Figure 8:**
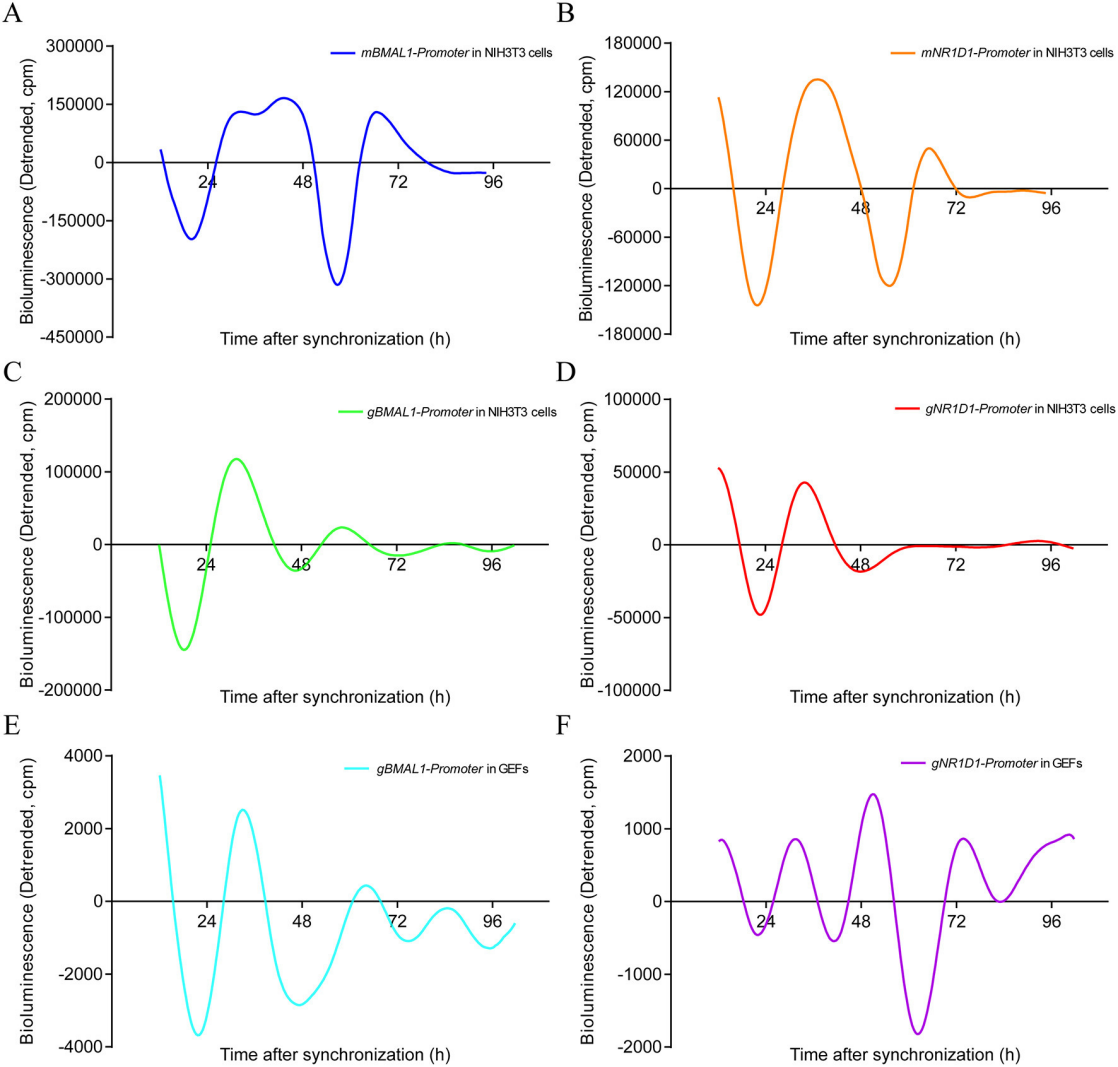
Real-time luciferase assays in NIH3T3 cells or GEFs with two different promoters of mouse and goat. Representative records of bioluminescence from NIH3T3 cells transfected with pGL4.22-mBMAL1-Promoter-*Luc* **(A)** or pGL4.22-mNR1D1-Promoter-*Luc* **(B)**. Representative records of bioluminescence from NIH3T3 cells transfected with pGL4.22-gBMAL1-Promoter-*Luc* **(C)** or pGL4.22-gNR1D1-Promoter-*Luc* **(D)**. Representative records of bioluminescence from GEFs transfected with pGL4.22-gBMAL1-Promoter-*Luc* **(E)** or pGL4.22-gNR1D1-Promoter-*Luc* **(F)**. Representative results from three independent repetitions are shown. GEFs, goat embryonic fibroblasts.

To further confirm the existence of circadian oscillators in goats, the *BMAL1* and *NR1D1* promoter luciferase reporter vectors of mouse and goat were transfected into NIH3T3 fibroblasts, which are a valuable platform for studying mammalian circadian pacemakers *in vitro* because of their self-sustained robust circadian rhythms in clock gene expression (44). As expected, pGL4.22-mBMAL1-Promoter*-Luc* and pGL4.22-mNR1D1-Promoter-*Luc* in NIH3T3 cells displayed rhythmic oscillations with periods of approximately 24 h after DXM synchronization (Figures 8A,B). Notably, pGL4.22-gBMAL1-Promoter*-Luc* and pGL4.22-gNR1D1-Promoter-*Luc* in NIH3T3 cells also displayed rhythmic oscillations with periods of approximately 24 h after DXM synchronization (Figures 8C,D). Furthermore, when pGL4.22-gBMAL1-Promoter-*Luc* and pGL4.22-gNR1D1-Promoter-*Luc* were transfected into GEFs, all constructs also displayed rhythmic oscillations with near-24-h periods after DXM synchronization (Figures 8E,F). Notably, whether in NIH3T3 cells or GEFs, the *BMAL1* gene reporters both exhibited the higher amplitudes than *NR1D1*. It is worth mentioning that the amplitude of g*BMAL1-Luc* and *gNR1D1-Luc* expression in NIH3T3 cells was higher than that in GEFs, whereas m*BMAL1-Luc* and m*NR1D1-Luc* expression in NIH3T3 cells had the highest amplitude. These data further confirm the existence of a functional circadian clock system in goats.

## Discussion

The circadian clock system coordinates the operation of all parts of the body among various mammalian cells, tissues, and organs to ensure that the physiological functions, biochemical processes, and behaviors of the body are coherent (45). Since the end of the 20th century, with the development of molecular biology techniques, mammalian clock genes have been cloned frequently, and the molecular mechanisms of the circadian clock system have been gradually dissected by numerous researchers, attracting increased attention to the role of the circadian clock system in regulating various physiological functions (46). Owing to the convenience of obtaining genetic information and the relative ease of transgene construction, targeted gene deletion, and genome-wide random mutation screening, mouse models have become a frontier of circadian clock system studies. However, it is unclear whether the circadian clock system is conserved in different species of mammals, such as ruminants.

Goats, being one of the earliest domesticated livestock, play an important role in human agricultural civilization and economic development and have been the focus of animal husbandry and veterinary medicine. Previous studies have reported that serum testosterone levels of male goats change seasonally (47). In addition, a previous study has also demonstrated that the daily ambient temperature cycle can synchronize the central clock of goats by entraining body temperature, locomotor activity, and plasma melatonin rhythms (48). A recent study demonstrated that rhythmic expression of clock genes is present in the whole blood of goats (49). A recent study by our group also found that the clock gene *BMAL1* contributes to testosterone production by regulating the transcription of steroidogenic genes in goat Leydig cells, providing novel evidence for the important role of the circadian clock system in regulating male ruminant reproduction (22). However, the specific molecular mechanisms of the goat circadian clock system remain largely unknown. In the current study, the molecular mechanism of the goat circadian clock system was dissected to prove that the transcriptional regulatory mechanisms of the mammalian circadian clock system in goats and mice are conservative, and that the goat circadian oscillators are not as robust as those of mice, at least in the current experimental models.

In this study, GEFs were used as a cell model to investigate the goat circadian clock system *in vitro*. The tissue block attachment method with a simple operation and a high success rate was selected to isolate cells, and high-purity and high-vitality GEFs were identified through cell morphology observation and immunofluorescence staining of fibroblast-specific markers (Figure 1). Liver and kidney tissues were selected to investigate the goat circadian clock system *in vivo*. The findings revealed that the expression profiles of clock genes at the mRNA and protein levels exhibited a self-sustaining and robust circadian rhythm in GEFs, goat liver, and kidney tissues (Figures 2, 3C–F, 4, 5). Notably, *NR1D1* gene expression was the lowest when *BMAL1* gene expression reached its peak in GEFs, which showed that the transcript level of *NR1D1* was in anti-phase with *BMAL1* (Figure 2). This is consistent with observations from goat Leydig cells (22) as well as those from mice, rats, and humans (50–52). However, the antiphase of BMAL1 and NR1D1 at the protein level was not evident in GEFs (Figures 3C–F). This observation is inconsistent with a previous report on human chondrocytes, which showed nearly opposite phases of BMAL1 and NR1D1 proteins (50). The antiphase of *BMAL1* and *NR1D1* at the mRNA and protein levels was evident in goat liver and kidney tissues (Figures 4, 5), which is consistent with a previous report in mice (53, 54). In addition, the expression profiles of *CRY1* and *CRY2* in goat liver and kidney tissues were consistent with previous reports in goat whole blood, showing low expression at 10:00 h and high expression at 22:00 h (49). However, the expression profiles of *PER2* and *PER3* in goat liver and kidney tissues were inconsistent with a previous report in goat whole blood (49). We speculate that this discrepancy may be caused by the phase shift of the goat circadian clock owing to photoperiod differences; however, the specific reasons require further investigation.

In this study, our data also revealed that the amino acid sequences of goat circadian clock proteins are highly homologous to those of cows, mice, and humans (Table 3). In addition, the tertiary structure of goat BMAL1 and CLOCK protein monomers is similar to that of mice and humans, both containing bHLH-PAS domains, and the RMSD values obtained by pairwise comparison were all less than 0.06 (Figure 6). The tertiary structures of the goat, mouse, and human BMAL1/CLOCK heterodimers were also highly similar (Figure 6). Because the function of a protein depends on its structure, it was hypothesized that goat, mouse, and human BMAL1/CLOCK dimers might also have similar functions. The BMAL1 and CLOCK proteins in goats may be critical for the regulation of the caprine circadian clock system. In addition, a large number of RORE *cis*-acting elements were found in the proximal promoter region of the goat clock gene, *BMAL1* (Figure 7A; Table 4). Similarly, the goat clock gene *NR1D1* was found to be a perfect hybrid promoter gene with both RORE and E-box *cis*-acting elements in its promoter region (Figure 7A; Table 4). This is consistent with previously reported observations in mice and sheep (55) and provides theoretical support for research on the transcriptional and translational regulatory mechanisms of the goat circadian clock system.

To detect whether the molecular regulation mechanisms of the circadian clock system in goats and mice are conserved, the transcription factors of goats and mice were used to perform dual-luciferase reporter assays and real-time monitoring of bioluminescence, respectively. Dual-luciferase reporter assays showed that gRORα could activate the promoter activity of the goat *BMAL1* gene, while gNR1D1 repressed it (Figure 7B). The promoter activity of goat *NR1D1* was activated by gBMAL1/gCLOCK or mBMAL1/mCLOCK, whereas it was repressed by the addition of gCRY2 or mPER2 (Figure 7C). Real-time bioluminescence assays also revealed that the transcriptional activities of *BMAL1* and *NR1D1* in goats and mice exhibited rhythmic changes over a period of approximately 24 h in NIH3T3 cells or GEFs (Figure 8). Both results suggest a high degree of conservation between goats and mice. However, it must be mentioned that the increase in luciferase activity induced by goat BMAL1/CLOCK on the E-box-driven promoter luciferase reporter vector was much less than that by mice (Fig 5C). We speculate that this functional difference may be caused by the different regulatory regions, which are covered to varying degrees and formed by the subtle differences between goat and mouse BMAL1/CLOCK transcription factor dimers (Fig 4, RMSD = 3.416). Real-time bioluminescence results also showed that g*BMAL1-Luc* and g*NR1D1-Luc* expression had higher amplitude in NIH3T3 mouse fibroblasts than those in GEFs (Figure 8), which may be caused by the different transfection efficiencies between the two cells. However, m*BMAL1-Luc* and m*NR1D1-Luc* expression in NIH3T3 cells had the highest amplitude, which was consistent with the results of the dual-luciferase reporter assay. Therefore, it is reasonable to conclude that the circadian clock system of goats is not as robust as that of mice, at least in the current experimental models; however, the specific reasons require further study.

In conclusion, the above data suggest that several salient transcriptional features established using mouse clock components can be reproduced using goat clock protein and gene promoter luciferase reporter vectors. Therefore, the molecular clock mechanisms described in goat fibroblasts share a high degree of similarity with the current mammalian circadian clock model (6). However, the rhythmic oscillations of the circadian clock system of goats are not as robust as those of mice, at least in the current experimental models. These data provide a reliable basis for further understanding the involvement of the circadian clock system in regulating the physiological functions of goats.

## Data Availability Statement

The original contributions presented in the study are included in the article/supplementary material, further inquiries can be directed to the corresponding author.

## Ethics Statement

The animal study was reviewed and approved by all animal procedures were approved under the control of the Guidelines for Animal Experiments by the Committee for the Ethics on Animal Care and Experiments of Northwest University (Approval No. 2021028) and performed under the control of the Guidelines on Ethical Treatment of Experimental Animals (2006) No. 398 set by the Ministry of Science and Technology, China. Written informed consent was obtained from the owners for the participation of their animals in this study.

## Author Contributions

HC and DG: designed the experiments, prepared figures, and drafted the manuscript. DG, HC, HoZ, HD, YL, and JZ: performed the experiments. HC, DG, HaZ, YZ, HJ, and XW: analyzed the data. HC, DG, and AW: interpreted the results of the experiments. HC and YJ: supervised the project and edited the manuscript. All authors approved the final version of this manuscript.

## Funding

This work was supported by the National Natural Science Foundation of China Grants 31771301 (to HC) and 31602125 (to HC) and the Agricultural Science Foundation of Shaanxi Province Grants NYKJ-2021-YL (XN) 10 (to HC).

## Conflict of Interest

The authors declare that the research was conducted in the absence of any commercial or financial relationships that could be construed as a potential conflict of interest.

## Publisher's Note

All claims expressed in this article are solely those of the authors and do not necessarily represent those of their affiliated organizations, or those of the publisher, the editors and the reviewers. Any product that may be evaluated in this article, or claim that may be made by its manufacturer, is not guaranteed or endorsed by the publisher.

## Abbreviations

GEF: goat embryonic fibroblast
BMAL1/ARNTL: aryl hydrocarbon receptor nuclear translocator-like protein 1
DBP: D site albumin promoter binding protein
NFIL3/E4BP4: nuclear factor interleukin 3-regulated protein
NR1D1/REV-ERBα: nuclear receptor subfamily 1 group D member 1
NR1D2/REV-ERBβ: nuclear receptor subfamily 1 group D member 2
RORα: retinoid-related orphan receptor alpha
CRY: cryptochrome
CLOCK: circadian locomotor output cycles kaput
PER: period
SCN: suprachiasmatic nucleus
RORE: retinoic acid-related orphan receptor response element
qPCR: real-time quantitative PCR
WB: western blotting
PBS: phosphate-buffered saline
FBS: fetal bovine serum
AA: antibiotic-antimycotic
DXM: dexamethasone
HRP: horseradish peroxidase
CDS: coding sequences
NCBI: National Center for Biotechnology Information
DMEM: Dulbecco's Modified Eagle Medium
RMSD: root mean squared deviation
Luc: luciferase.

## References

[1] Bass J Takahashi JS. Circadian integration of metabolism and energetics. Science. (2010) 330:1349–54. 10.1126/science.1195027 21127246 PMC3756146

[2] Tahara Y Shibata S. Chrono-biology, chrono-pharmacology, and chrono-nutrition. J Pharmacol Sci. (2014) 124:320–35. 10.1254/jphs.13R06CR 24572815

[3] Dvornyk V Vinogradova O Nevo E. Origin and evolution of circadian clock genes in prokaryotes. Proc Natl Acad Sci U S A. (2003) 100:2495–500. 10.1073/pnas.0130099100 12604787 PMC151369

[4] Roenneberg T Merrow M. The circadian clock and human health. Curr Biol. (2016) 26:R432–43. 10.1016/j.cub.2016.04.011 27218855

[5] Chaix A Zarrinpar A Panda S. The circadian coordination of cell biology. J Cell Biol. (2016) 215:15–25. 10.1083/jcb.201603076 27738003 PMC5057284

[6] Cox KH Takahashi JS. Circadian clock genes and the transcriptional architecture of the clock mechanism. J Mol Endocrinol. (2019) 63:R93–R102. 10.1530/JME-19-0153 31557726 PMC6872945

[7] Buhr ED Takahashi JS. Molecular components of the Mammalian circadian clock. Handb Exp Pharmacol. (2013) 217:3–27. 10.1007/978-3-642-25950-0_1 23604473 PMC3762864

[8] Duong HA Robles MS Knutti D Weitz CJ. A molecular mechanism for circadian clock negative feedback. Science. (2011) 332:1436–9. 10.1126/science.1196766 21680841 PMC3859310

[9] Ko CH Takahashi JS. Molecular components of the mammalian circadian clock. Hum Mol Genet. (2006) 15 Spec No 2: R271–7. 10.1093/hmg/ddl207 16987893

[10] Aryal RP Kwak PB Tamayo AG Gebert M Chiu PL Walz T. Macromolecular assemblies of the mammalian circadian clock. Mol Cell. (2017) 67:770–82 e6. 10.1016/j.molcel.2017.07.017 28886335 PMC5679067

[11] Richards J Gumz ML. Mechanism of the circadian clock in physiology. Am J Physiol Regul Integr Comp Physiol. (2013) 304:R1053–64. 10.1152/ajpregu.00066.2013 23576606 PMC4073891

[12] Preitner N Damiola F Lopez-Molina L Zakany J Duboule D Albrecht U. The orphan nuclear receptor REV-ERBalpha controls circadian transcription within the positive limb of the mammalian circadian oscillator. Cell. (2002) 110:251–60. 10.1016/S0092-8674(02)00825-5 12150932

[13] Guillaumond F Becquet D Boyer B Bosler O Delaunay F Franc JL. DNA microarray analysis and functional profile of pituitary transcriptome under core-clock protein BMAL1 control. Chronobiol Int. (2012) 29:103–30. 10.3109/07420528.2011.645707 22324551

[14] Liu AC Tran HG Zhang EE Priest AA Welsh DK Kay SA. Redundant function of REV-ERBalpha and beta and non-essential role for Bmal1 cycling in transcriptional regulation of intracellular circadian rhythms. PLoS Genet. (2008) 4:e1000023. 10.1371/journal.pgen.1000023 18454201 PMC2265523

[15] Eide EJ Woolf MF Kang H Woolf P Hurst W Camacho F. Control of mammalian circadian rhythm by CKI epsilon-regulated proteasome-mediated PER2 degradation. Mol Cell Biol. (2005) 25:2795–807. 10.1128/MCB.25.7.2795-2807.2005 15767683 PMC1061645

[16] Stratmann M Suter DM Molina N Naef F Schibler U. Circadian Dbp transcription relies on highly dynamic BMAL1-CLOCK interaction with E boxes and requires the proteasome. Mol Cell. (2012) 48:277–87. 10.1016/j.molcel.2012.08.012 22981862

[17] Duez H Van Der Veen JN Duhem C Pourcet B Touvier T Fontaine C. Regulation of bile acid synthesis by the nuclear receptor Rev-erb alpha. Gastroenterology. (2008) 135:689–98. 10.1053/j.gastro.2008.05.035 18565334

[18] Ferreira ACF Szeto ACH Heycock MWD Clark PA Walker JA Crisp A. ROR alpha is a critical checkpoint for T cell and ILC commitment in the embryonic thymus. Nat Immunol. (2021) 22:166–U106. 10.1038/s41590-020-00833-w 33432227 PMC7116838

[19] Yoshitane H Asano Y Sagami A Sakai S Suzuki Y Okamura H. Functional D-box sequences reset the circadian clock and drive mRNA rhythms. Commun Biol. (2019) 2. 10.1038/s42003-019-0522-3 31428688 PMC6687812

[20] Li Y Ma J Yao K Su W Tan B Wu X. Circadian rhythms and obesity: Timekeeping governs lipid metabolism. J Pineal Res. (2020) 69:e12682. 10.1111/jpi.12682 32656907

[21] Kettner NM Mayo SA Hua J Lee C Moore DD Fu L. Circadian dysfunction induces leptin resistance in mice. Cell Metab. (2015) 22:448–59. 10.1016/j.cmet.2015.06.005 26166747 PMC4558341

[22] Xiao Y Zhao L Li W Wang X Ma T Yang L. Circadian clock gene BMAL1 controls testosterone production by regulating steroidogenesis-related gene transcription in goat Leydig cells. J Cell Physiol. (2021) 236:6706–25. 10.1002/jcp.30334 33598947

[23] Li C Zhang L Ma T Gao L Yang L Wu M. Bisphenol A attenuates testosterone production in Leydig cells via the inhibition of NR1D1 signaling. Chemosphere. (2021) 263:128020. 10.1016/j.chemosphere.2020.128020 33297044

[24] Zhao L Yang L Zhang J Xiao Y Wu M Ma T. Bmal1 promotes prostaglandin E2 synthesis by upregulating Ptgs2 transcription in response to increasing estradiol levels in day 4 pregnant mice. Am J Physiol Endocrinol Metab. (2021) 320:E747–59. 10.1152/ajpendo.00466.2020 33554778

[25] Lee JH Sancar A. Circadian clock disruption improves the efficacy of chemotherapy through p73-mediated apoptosis. Proc Natl Acad Sci U S A. (2011) 108:10668–72. 10.1073/pnas.1106284108 21628572 PMC3127903

[26] Yang X Wood PA Oh EY Du-Quiton J Ansell CM Hrushesky WJ. Down regulation of circadian clock gene period 2 accelerates breast cancer growth by altering its daily growth rhythm. Breast Cancer Res Treat. (2009) 117:423–31. 10.1007/s10549-008-0133-z 18651214

[27] Oishi Y Hayashi S Isagawa T Oshima M Iwama A Shimba S. Bmal1 regulates inflammatory responses in macrophages by modulating enhancer RNA transcription. Sci Rep. (2017) 7:7086. 10.1038/s41598-017-07100-3 28765524 PMC5539165

[28] Selcho M Millan C Palacios-Munoz A Ruf F Ubillo L Chen J. Central and peripheral clocks are coupled by a neuropeptide pathway in Drosophila. Nat Commun. (2017) 8:15563. 10.1038/ncomms15563 28555616 PMC5459987

[29] Rehman A Ahmad E Arshad U Riaz A Akhtar MS Ahmad T. Effects of immunization against inhibin alpha-subunit on ovarian structures, pregnancy rate, embryonic and fetal losses, and prolificacy rate in goats where estrus was induced during the non-breeding season. Anim Reprod Sci. (2021) 224:106654. 10.1016/j.anireprosci.2020.106654 33249352

[30] El Allali K Farsi H Piro M Achaban MR Ouassat M Challet E. Smartphone and a freely available application as a new tool to record locomotor activity rhythm in large mammals and humans. Chronobiol Int. (2019) 36:1047–57. 10.1080/07420528.2019.1609980 31088178

[31] Piccione G Giannetto C Assenza A Fazio F Caola G. Locomotor activity and serum tryptophan and serotonin in goats: daily rhythm. J Appl Biomed. (2008) 6:73–9. 10.32725/jab.2008.010

[32] Farsi H Harti D Achaaban MR Piro M Ouassat M Challet E. Validation of locomotion scoring as a new and inexpensive technique to record circadian locomotor activity in large mammals. Heliyon. (2018) 4:e00980. 10.1016/j.heliyon.2018.e00980 30582033 PMC6287081

[33] Chen H Gao L Xiong Y Yang D Li C Wang A. Circadian clock and steroidogenic-related gene expression profiles in mouse Leydig cells following dexamethasone stimulation. Biochem Biophys Res Commun. (2017) 483:294–300. 10.1016/j.bbrc.2016.12.149 28025148

[34] Chen H Gao L Yang D Xiao Y Zhang M Li C. Coordination between the circadian clock and androgen signaling is required to sustain rhythmic expression of Elovl3 in mouse liver. J Biol Chem. (2019) 294:7046–56. 10.1074/jbc.RA118.005950 30862677 PMC6497949

[35] Gao L Chen H Li C Xiao Y Yang D Zhang M. ER stress activation impairs the expression of circadian clock and clock-controlled genes in NIH3T3 cells via an ATF4-dependent mechanism. Cell Signal. (2019) 57:89–101. 10.1016/j.cellsig.2019.01.008 30703445

[36] Chen H Zhao L Chu G Kito G Yamauchi N Shigeyoshi Y. FSH induces the development of circadian clockwork in rat granulosa cells via a gap junction protein Cx43-dependent pathway. Am J Physiol Endocrinol Metab. (2013) 304:E566–75. 10.1152/ajpendo.00432.2012 23299500

[37] Yang L Ma T Zhao L Jiang H Zhang J Liu D. Circadian regulation of apolipoprotein gene expression affects testosterone production in mouse testis. Theriogenology. (2021) 174:9–19. 10.1016/j.theriogenology.2021.06.023 34416563

[38] Zhao L Zhang J Yang L Zhang H Zhang Y Gao D. Glyphosate exposure attenuates testosterone synthesis via NR1D1 inhibition of StAR expression in mouse Leydig cells. Sci Total Environ. (2021) 785:147323. 10.1016/j.scitotenv.2021.147323 33957581

[39] Chen H Chu G Zhao L Yamauchi N Shigeyoshi Y Hashimoto S. Rev-erbalpha regulates circadian rhythms and StAR expression in rat granulosa cells as identified by the agonist GSK4112. Biochem Biophys Res Commun. (2012) 420:374–9. 10.1016/j.bbrc.2012.02.164 22425774

[40] Zhao L Xiao Y Li C Zhang J Zhang Y Wu M. Zearalenone perturbs the circadian clock and inhibits testosterone synthesis in mouse Leydig cells. J Toxicol Environ Health A. (2021) 84:112–24. 10.1080/15287394.2020.1841699 33148124

[41] Nelson W Tong YL Lee JK Halberg F. Methods for cosinor-rhythmometry. Chronobiologia. (1979) 6:305–23. 548245

[42] Coulombe PA Wong P. Cytoplasmic intermediate filaments revealed as dynamic and multipurpose scaffolds. Nat Cell Biol. (2004) 6:699–706. 10.1038/ncb0804-699 15303099

[43] Kirchmair J Markt P Distinto S Wolber G Langer T. Evaluation of the performance of 3D virtual screening protocols: RMSD comparisons, enrichment assessments, and decoy selection–what can we learn from earlier mistakes? J Comput Aided Mol Des. (2008) 22:213–28. 10.1007/s10822-007-9163-6 18196462

[44] Akashi M Nishida E. Involvement of the MAP kinase cascade in resetting of the mammalian circadian clock. Genes Dev. (2000) 14:645–9. 10.1101/gad.14.6.645 10733524 PMC316464

[45] Partch CL Green CB Takahashi JS. Molecular architecture of the mammalian circadian clock. Trends Cell Biol. (2014) 24:90–9. 10.1016/j.tcb.2013.07.002 23916625 PMC3946763

[46] Turek FW. Circadian clocks: Not your grandfather's clock. Science. (2016) 354:992–3. 10.1126/science.aal2613 27885003

[47] Howland BE Sanford LM Palmer WM. Changes in serum levels of LH, FSH, prolactin, testosterone, and cortisol associated with season and mating in male pygmy goats. J Androl. (1985) 6:89–96. 10.1002/j.1939-4640.1985.tb00822.x 3921507

[48] Farsi H Harti D Achaaban MR Piro M Raverot V Bothorel B. Melatonin rhythm and other outputs of the master circadian clock in the desert goat (Capra hircus) are entrained by daily cycles of ambient temperature. J Pineal Res. (2020) 68:e12634. 10.1111/jpi.12634 32011000

[49] Giannetto C Cannella V Giudice E Guercio A Arfuso F Piccione G. Clock genes determination in whole blood in goats housed under a long light cycle. Chronobiol Int. (2021) 38:1283–9. 10.1080/07420528.2021.1928158 34000942

[50] Akagi R Akatsu Y Fisch KM Alvarez-Garcia O Teramura T Muramatsu Y. Dysregulated circadian rhythm pathway in human osteoarthritis: NR1D1 and BMAL1 suppression alters TGF-beta signaling in chondrocytes. Osteoarthritis Cartilage. (2017) 25:943–51. 10.1016/j.joca.2016.11.007 27884645 PMC5438901

[51] Rath MF Rohde K Fahrenkrug J Moller M. Circadian clock components in the rat neocortex: daily dynamics, localization and regulation. Brain Struct Funct. (2013) 218:551–62. 10.1007/s00429-012-0415-4 22527123

[52] Gossan N Zeef L Hensman J Hughes A Bateman JF Rowley L. The circadian clock in murine chondrocytes regulates genes controlling key aspects of cartilage homeostasis. Arthritis Rheum. (2013) 65:2334–45. 10.1002/art.38035 23896777 PMC3888512

[53] Dufour CR Levasseur MP Nguyen HHP Eichner LJ Wilson BJ Charest-Marcotte A. Genomic convergence among ERR alpha, PROX1, and BMAL1 in the control of metabolic clock outputs. Plos Genetics. (2011) 7:e1002143. 10.1371/journal.pgen.1002143 21731503 PMC3121748

[54] Valcin JA Udoh US Swain TM Andringa KK Patel CR Al Diffalha S. Alcohol and liver clock disruption increase small droplet macrosteatosis, alter lipid metabolism and clock gene mRNA rhythms, and remodel the triglyceride lipidome in mouse liver. Front Physiol. (2020) 11:1048. 10.3389/fphys.2020.01048 33013449 PMC7504911

[55] Dardente H Fustin JM Hazlerigg DG. Transcriptional feedback loops in the ovine circadian clock. Comp Biochem Physiol A Mol Integr Physiol. (2009) 153:391–8. 10.1016/j.cbpa.2009.03.016 19341811

